# Polyamidoamine Dendrimers: Brain-Targeted Drug Delivery Systems in Glioma Therapy

**DOI:** 10.3390/polym16142022

**Published:** 2024-07-15

**Authors:** Xinyi Yan, Qi Chen

**Affiliations:** 1 Key Laboratory of Neuropharmacology and Translational Medicine of Zhejiang Province, School of Pharmaceutical Sciences, Zhejiang Chinese Medical University, Hangzhou 310053, China; 13055425088@163.com; 2 Interdisciplinary Institute for Medical Engineering, Fuzhou University, Fuzhou 350108, China

**Keywords:** PAMAM dendrimers, brain-targeted, drug delivery system, glioma

## Abstract

Glioma is the most common primary intracranial tumor, which is formed by the malignant transformation of glial cells in the brain and spinal cord. It has the characteristics of high incidence, high recurrence rate, high mortality and low cure rate. The treatments for glioma include surgical removal, chemotherapy and radiotherapy. Due to the obstruction of the biological barrier of brain tissue, it is difficult to achieve the desired therapeutic effects. To address the limitations imposed by the brain’s natural barriers and enhance the treatment efficacy, researchers have effectively used brain-targeted drug delivery systems (DDSs) in glioma therapy. Polyamidoamine (PAMAM) dendrimers, as branched macromolecular architectures, represent promising candidates for studies in glioma therapy. This review focuses on PAMAM-based DDSs in the treatment of glioma, highlighting their physicochemical characteristics, structural properties as well as an overview of the toxicity and safety profiles.

## 1. Introduction

Dendrimers have been progressively developed since the 1980s [1]. Dendrimers, characterized by their highly branched, dendritic architecture, typically display a symmetric, core-centric organization and adopt a spherical, three-dimensional conformation when dispersed in aqueous environments. Due to their well-organized structure, in recent years, functionalized dendrimers have been continuously synthesized by researchers, positioning them as compelling candidates for drug delivery applications. Drug molecules can be attached to or embedded in the internal cavities of dendrimers, where numerous branches intertwine to form a hydrophobic cavity.

**Figure 1:**
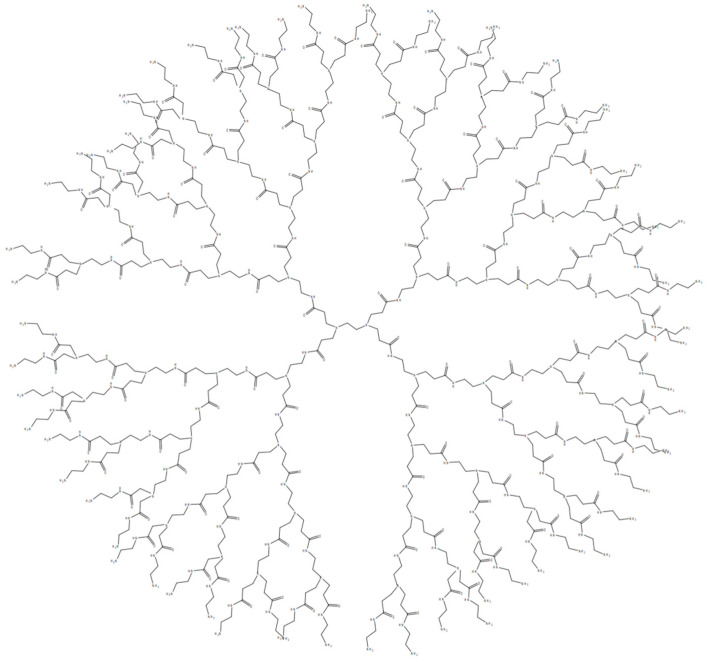
Structure of fourth-generation PAMAM dendrimer. Reprinted with the permission of [15].

One of the dendrimers that has achieved the greatest utilization and success is polyamidoamine (PAMAM) [2]. PAMAM dendrimers are characterized by an internally activated core, multiple generations of branching units and terminal functional groups (Figure 1). Amines are fundamental building blocks of the PAMAM dendritic architecture. Notable examples include ethylenediamine (EDA), diaminobutane (DAB) and 1,6-diaminohexane (DAH) [3,4]. The properties of PAMAM can be further enhanced by customizable functional groups, enabling conjugation with a wide array of therapeutic molecules [5,6,7]. Thus, via modification, PAMAM-based drug delivery systems (DDSs) exhibit many outstanding properties, including low immunogenicity, high water solubility, good biodegradability, excellent biocompatibility, a very low non-specific plasma protein binding capacity, precisely controlled drug release properties, and so on [8,9,10]. PAMAM-based DDSs can be traced back to a pioneering study in 1995, where researchers proposed the employment of PAMAM dendrimers as vehicles for DNA delivery [11]. Then, this study sparked immense interest, propelling the exploration of PAMAM-based DDSs in a broad spectrum of diseases, especially for various cancers. In 2012, Liu and colleagues fabricated a fifth-generation PAMAM dendrimer (G5) with a triethanolamine (TEA) core to effectively transport siRNAs adorned with complementary A(n)/T(n) sequences at their 3’ ends within a prostate cancer model [12]. The siRNA delivery system achieved a remarkable downregulation of heat shock protein 27 (Hsp27) gene expression via RNA interference, ultimately yielding obvious anti-tumor effects. In 2016, a study conducted by Oddone et al. examined the internalization of PAMAM G4.5 carboxylic acid dendrimers in 4T1 breast cancer cells [13]. In the same year, Gao et al. designed a gene delivery system for glioma using a transferrin (Tf)-modified PAMAM dendrimer [14]. Their results showed that the transferrin (Tf)-modified PAMAM displayed improved targeting capability in homing to glioma cells when compared to non-modified PAMAM.

This review primarily focuses on the applications of PAMAM in glioma, covering studies conducted from 1995 to 2023. Glioma, a malignant tumor frequently encountered in the central nervous system (CNS), is marked by the rapid proliferation of cancerous glial cells. It comprises approximately half of all brain tumors, with an annual incidence varying between 30 and 80 cases per million individuals [16,17]. The standard treatment for gliomas begins with surgery to remove the tumor, which may be followed by radiation or chemotherapy to consolidate the efficacy, depending on the patient’s condition [18]. In the last two decades, chemotherapeutics have evolved from agents like carmustine, nimustine and lomustine to temozolomide (TMZ) as the preferred first-line therapy. However, despite the combination of TMZ with radiotherapy and surgical intervention, the resulting improvement in long-term survival rates beyond five years has been marginal [19,20]. The inherent challenge is the resistance of glioma cells to chemotherapy and radiotherapy. Additionally, there exist the following two important biological barriers: the blood–brain barrier (BBB) and the blood–brain tumor barrier. The presence of these two barriers greatly limits the ability of almost all macromolecular drugs and 98% of small-molecule drugs to enter the tumor region [21,22]. Therefore, researchers continue to strive for more precise and effective targeted therapy. Through rational design, PAMAM, a unique stellate dendritic molecule, can exhibit remarkable properties [23], such as a flexible molecular size, functionally rich molecular structure and the ability to penetrate the BBB. These advantages make PAMAM-based DDSs stand out in studies of glioma treatment. When compared to amphiphilic poly(N-vinyl-2-pyrrolidone), a linear PVP, which can also be used as a carrier for glioma therapy, PAMAM performed better in cellular uptake [24]. Sharma et al. systematically altered the PAMAM surface by selectively attaching glucose, mannose or galactose, and observed intriguing variations in their targeting capabilities [1]. They found that PAMAM modified with glucose significantly improved their affinity for tumor-associated macrophages (TAMs) and microglia, enhancing both their penetration into the brain and the rate of cellular uptake. The galactose-modified PAMAM exhibited a change in target specificity, redirecting its affinity from TAMs to show a preferred binding to galectins on glioblastoma cell membranes. Notably, the mannose-modified PAMAM maintained PAMAM’s original specificity towards TAMs and microglia without altering the targeting profile. Nevertheless, they displayed alterations in the kinetic patterns of the accumulation within gliomas. Ortiz and colleagues used PAMAM for the effective transportation of methotrexate to U87 glioma cells [25]. Their results showed that the formulation with PAMAM elevated the cytotoxic efficacy of methotrexate against U87 glioma cells in comparison to the administration of free methotrexate. Furthermore, they demonstrated that fourth-generation PAMAM with 25% acetylation played a pivotal role in augmenting the absorption of the drug by glioma cells.

Additionally, this review briefly discusses the fundamental characteristics of PAMAM and the methodologies employed in their synthesis, and addresses concerns about their toxicity and overall safety profiles. It further provides an overview of how PAMAM-based DDSs have evolved through purposeful adjustments. By incorporating genes, custom-designed ligands, cell-penetrating peptides and other elements, these PAMAM-based DDSs can be engineered to selectively target both brain tissue and specific tumor sites with remarkable precision. The engineering not only boosts the efficacy of drug delivery but also enables a higher degree of targeting precision, thereby optimizing therapeutic strategies and holding significant promise for enhancing patient outcomes and prognosis. This review will facilitate the understanding of the complexities and prospects of PAMAM-based DDSs in glioma treatments.

Differing from other similar reviews that broadly emphasize the medical applications of PAMAM dendrimers, this review specifically focuses on the potential of PAMAM dendrimers in glioma therapy, underscoring their pivotal role in modern neuro-oncology. Beyond merely outlining PAMAM’s role, this review explores the progressive advancements in PAMAM-based nanotechnologies, illustrating how purposeful modifications involving genes, ligands, etc., have transformed these platforms into high-efficiency brain delivery systems.

## 2. Characteristics of PAMAM Dendrimers

Unlike linear polymers, which often exhibit a random distribution of molecular weights leading to polydispersity [26], PAMAMs display monodispersity, characterized by an identical size and structural uniformity [27]. PAMAM dendrimers have perfectly branched and tree-like configurations. Each generation of the dendrimer is built through a stepwise process where new branches are added in a controlled manner onto the existing structure. As a result, the size, shape, number and type of surface functional groups, as well as their overall physicochemical properties, including their charge, can be meticulously tailored during synthesis [28,29]. The monodispersity and structural precision of the PAMAM dendrimers enable them with several key advantages in biomedical applications:

1. Specific Targeting: Functional groups on the surface of PAMAM dendrimers can be chemically modified to attach targeting ligands, like transferrin, allowing selective binding to receptors on target cells [30].
2. Controlled Drug Release: Drugs can either be encapsulated within PAMAM dendrimers or attached to their surface, with drug release being manipulated by factors such as pH sensitivity, which facilitates targeted delivery and potentially reduces side-effects [31,32].
3. Improved Solubility: Hydrophobic drugs can be encapsulated within the interior of PAMAM, improving their solubility and bioavailability [33].
4. Reduced Toxicity: The clear structure minimizes the presence of potentially toxic byproducts, which are often associated with linear polymer degradation [34,35].
5. Enhanced Blood Stability: By conjugating polyethylene glycol (PEG) chains to PAMAM dendrimers, the circulating time of the dendrimers in the bloodstream is prolonged, thereby facilitating drug delivery to specific sites [36].

These properties make PAMAM dendrimers and similar dendritic polymers invaluable tools in drug delivery research, particularly in the development of advanced therapies for hard-to-reach areas like the brain. When compared to other nanoparticles (liposomes, micelles, silica, etc.), PAMAM has the smallest size [37], typically measuring within the 1 to 100 nanometer range. The small size is advantageous for their application in crossing biological barriers. Sarin and colleagues revealed the relationship between the size of functionalized dendrimers and their ability to cross the blood–brain tumor barrier in RG-2 malignant gliomas [38]. Specifically, they discovered that dendrimers with diameters below approximately 11.7 to 11.9 nanometers could successfully navigate through the BBTB’s pores, whereas larger dendrimers failed to do so. Generation 4 (G4) neutral PAMAM dendrimers, due to their neutral charge, minimize non-specific interactions. This characteristic enables them to evade rapid elimination by the immune system and prevents entrapment within the vasculature. Once in the CNS, these dendrimers can interact with specific cell types, such as microglia and astrocytes, which play critical roles in neuroinflammation and other neurological processes. By precisely targeting these cells, G4 neutral PAMAM dendrimers can be used to deliver therapeutic agents or modulate inflammatory responses directly at the site of action in the brain, which is especially beneficial for treating various brain diseases and disorders associated with inflammation, such as Alzheimer’s disease, multiple sclerosis, and brain injuries.

Additionally, the amine groups on the surface of PAMAM dendrimers are easily protonated in the tumor microenvironment. Under acidic conditions, PAMAM dendrimers usually releases drugs faster. Because of a high surface charge density and the proton sponge effect, dendritic molecules could be equipped with the capability of passive or/and active targeting of tumors [39,40]. PAMAM dendrimers have relatively stable physical and chemical properties, and the stability of PAMAM dendrimers can be altered by adjusting the pH and the concentration of the solution containing themselves [41,42]. Passive targeting of PAMAM dendrimers is achieved by constructing dendrimers with smaller particle sizes and by enhancing the permeability and retention (EPR) effect [43,44]. For example, Kukowska-Latallo et al. developed G5 PAMAM dendrimers, which were compact enough to evade the vascular system and enhance the EPR effect [45].

PAMAM dendrimers also can be used as surface activators [46]. PAMAM dendrimers incorporate lipophilic hydrocarbon chains alongside hydrophilic carboxyl and amino groups, endowing them with solubilizing, demulsifying and stabilizing capabilities akin to those exhibited by typical surfactants.

## 3. The Synthesis of PAMAM Dendrimers

The concept of “starburst polymers” was first proposed in 1985, now known as dendrimers [47]. The author also revealed the existence of PAMAM for the first time and described its synthesis process from the first generation to the seventh generation in detail. The commercialization of PAMAM has seen considerable success, and corresponding research and characterization efforts have attained a high level of maturity. Dendrimer synthesis encompasses numerous strategies, of which the convergent and divergent methods are prevalently employed [48]. The core unit of the initiator is gradually grown and polymerized in a layer-by-layer manner (represented by ‘generation’ or ‘G’).

**Figure 2:**
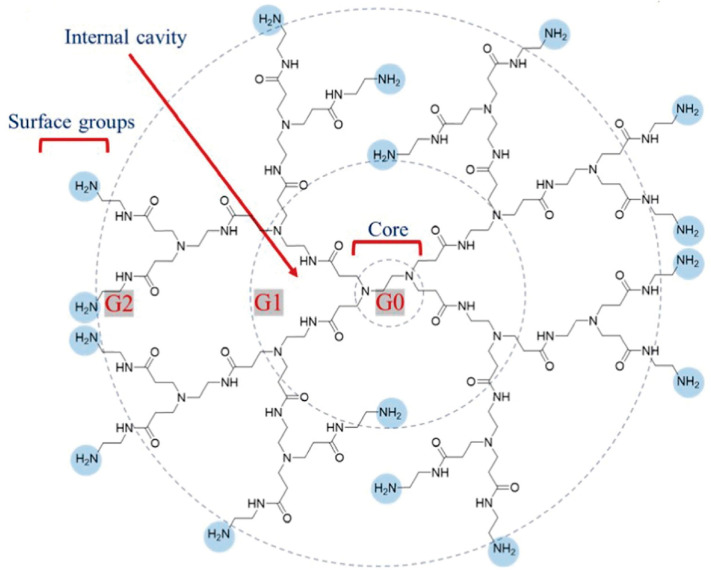
G0: The core of the PAMAM dendrimer, generation 0. G1: Cationic amino-terminated PAMAM dendrimer, generation 1. G2: Cationic amino-terminated PAMAM dendrimer, generation 2. PAMAM dendrimers are synthesized through a process known as divergent step-growth polymerization, which occurs in a sequential layer-by-layer fashion. This construction is quantified in terms of ‘generations’ (denoted as “G”), indicating the level of branching complexity from the core outwards. Reprinted with the permission of [3].

Nowadays, the main method for synthesizing PAMAM dendrimers is divergent synthesis (Figure 2). Initiating with ethylenediamine, half-generation PAMAM dendrimers are synthesized via a Michael addition reaction with methyl methacrylate. Then, the half-generation product is used to undergo reaction with excess ethylenediamine to obtain whole-generation PAMAM dendrimers. The above two reaction steps are repeated alternately to obtain PAMAM dendrimers with increasing generations [3]. As the generation number increases, both the diameter and molecular weight of PAMAM dendrimers experience steady augmentation.

In recent years, several innovative methodologies have emerged, encompassing techniques like convergent–divergent integration, click chemistry, hyper-core synthesis, branched monomer assembly, double-exponential reactions and LEGO-inspired chemistry [49,50,51].

## 4. Toxicity of PAMAM Dendrimers

Toxicity and safety considerations are paramount for PAMAM dendrimers. The toxicity to cells is influenced by factors such as the concentration, surface charge and other variables.

Cationic derivatives exhibit significantly higher toxicity compared to neutral or anionic derivatives [52,53,54]. The mechanism of PAMAM dendrimers through cells is mainly endocytosis and passive diffusion. Albertazzi et al. investigated the influence of dendrimer surface chemistry (cationic, neutral and hydrophobic/lipidated moieties), alongside their sizes of G2, 4 and 6, on the internalization mechanisms within cervical cancer (HeLa) cells [55]. The affinity of dendrimers for the cell membrane was observed to be dependent on their generation, which is reflected in the number of positive charges on their periphery, with G6 exhibiting the highest affinity followed by G4 and then G2. Studies have demonstrated that the toxicity of PAMAM dendrimers increases with larger generations. Furthermore, acetylation was found to decrease this affinity, while lipidation increased it [56].

PAMAM dendrimers with branched surfaces can be terminated by various functional groups, such as amino, carboxyl or hydroxyl groups. The choice and quantity of these terminal groups play a crucial role in determining the drug or ligand binding capacity and toxicity profile of PAMAM dendrimers. The amino-terminated PAMAM-NH_2_ exhibits a higher cytotoxicity compared to carboxyl-terminated PAMAM-COOH and hydroxyl-terminated PAMAM-OH. The functional groups on the surface of the whole-generation PAMAM are amino groups, and the functional groups on the surface of the half-generation PAMAM are carboxyl groups. Therefore, the cytotoxicity of the whole generation of PAMAM dendrimers is stronger than that of the half generation.

**Figure 3:**
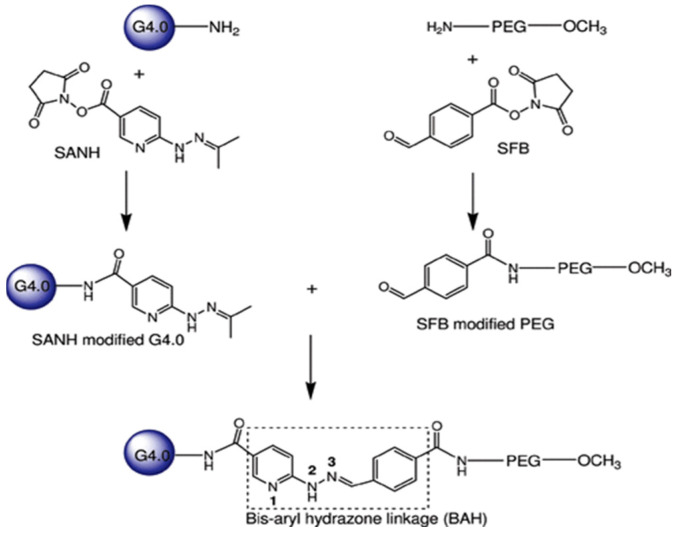
Surface modification of PAMAM dendrimers and their binding to polyethylene glycol (PEG). Reprinted with the permission of [61].

Although PAMAM dendrimers have important applications in pharmaceutical and biomedical fields, due to the cationic groups on the surface of PAMAM, they have considerable cytotoxicity, which limits their potential for clinical application [57]. Fortunately, there have been many methods to modify the surface groups of PAMAM to change their properties. Neutralizing the cationic groups of PAMAM by neutral or anionic functional groups can prevent the electrostatic interaction between PAMAM dendrimers and biomembranes, and help them to be non-toxic and biocompatible in the delivery of anticancer therapeutics [58]. For example, by conjugating polyethylene glycol (PEG) on the surface of PAMAM [59]. El-Sayed et al. used Caco-2 cells for examining the influence of surface modification on the cytotoxicity of PAMAM dendrimers [60]. Dendrimers were modified by conjugating either lauroyl chains or polyethylene glycol (PEG_2000_) onto the surface of cationic PAMAM dendrimers (G2, G3, and G4). A significant reduction in the cytotoxicity of cationic PAMAM dendrimers was observed when the surface was modified with the addition of six lauroyl or four PEG chains. This decrease in cytotoxicity is thought to be due to a reduction in/shielding of the positive charge on the dendrimer surface by the attached chains. Yuan et al. found that the incorporation of bis-aryl hydrazone (BAH) linkages into the vector (42 PEG chains per dendrimer) (Figure 3) significantly enhanced the buffering capacity [61]. The conjugates could complex with DNA plasmid tightly at low weight ratios and display dramatically improved cytocompatibility. Singh P et al. conjugated amine groups on fourth-generation PAMAM dendrimers with folic acid and folic acid-PEG- N-hydroxysuccinimide (NHS) [62]. The results demonstrated that folate-PEG-dendrimer conjugates exhibited a superior tumor-targeting efficiency and safety compared to non-PEGylated dendrimers. The integration of PEG-folic acid into the dendrimer structure reduced hemolytic toxicity, leading to a sustained drug release profile and optimized accumulation in the tumor region. The attachment of PEG chains onto dendrimer surfaces is influenced by factors such as chain length and the number of arms. These factors determine the capacity of dendrimers to encapsulate drugs within their cavities. Kojima et al. conducted a study where they encapsulated anticancer drugs within PEGylated dendrimers [63]. Their findings revealed that longer PEG arms lead to improved drug loading capabilities and stability.

The optimized PAMAM dendrimers successfully address toxicity concerns related to surface amino groups, while markedly improving their physical and chemical stability. This dual enhancement significantly boosts their biocompatibility, rendering them an optimal choice as DDSs for cancer.

## 5. PAMAM-Based Brain-Targeted DDSs

**Figure 4:**
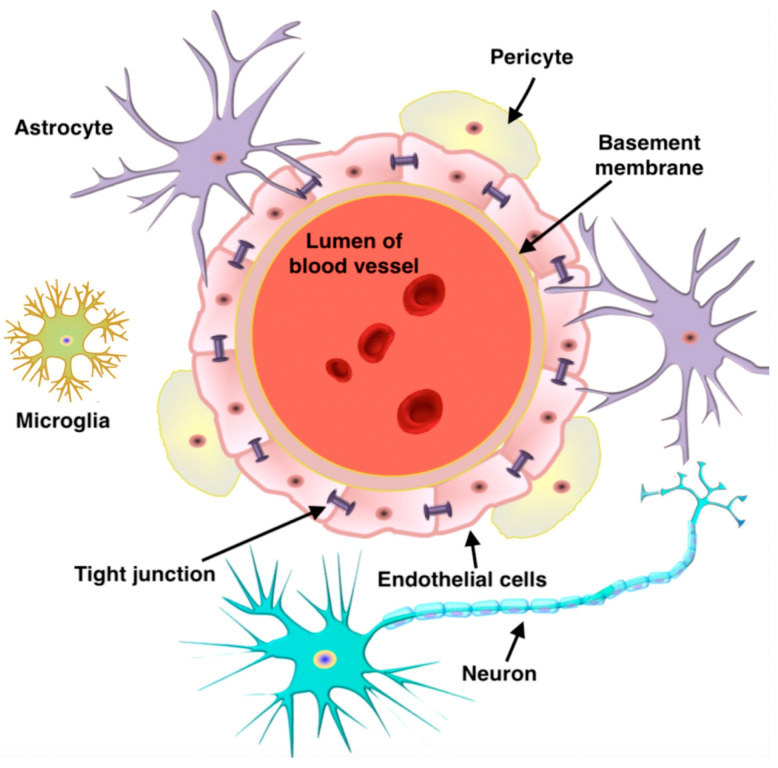
Schematic illustration of the structure of the BBB. Reprinted with the permission of [75].

Effectively crossing the BBB remains a central and challenging issue for the treatment of brain diseases [64,65,66]. The BBB represents a complex physiological isolation mechanism that regulates the exchange of substances between the blood and brain parenchyma [67,68,69]. The barrier is essential for maintaining the stability of the intracerebral environment, ensuring that neurons can function in a relatively constant, undisturbed environment. By restricting the entry of potentially harmful substances, toxins and certain macromolecules and pathogens from the bloodstream into the brain tissue, the BBB acts as a protective shield for the CNS, preventing external disturbances from affecting brain health and function [70,71,72]. This natural defense system is a key component of the brain’s self-protection mechanism and is fundamental to the normal functioning of the nervous system and overall physical health (Figure 4) [73,74,75]. However, the BBB also makes it difficult for many drug molecules, including large-molecule drugs and gene therapy vectors, to enter the brain tissue directly from the blood circulation, thus limiting their effectiveness in the treatment of a wide range of neurological disorders such as Alzheimer’s disease, Parkinson’s disease and brain tumors [76,77].

**Figure 5:**
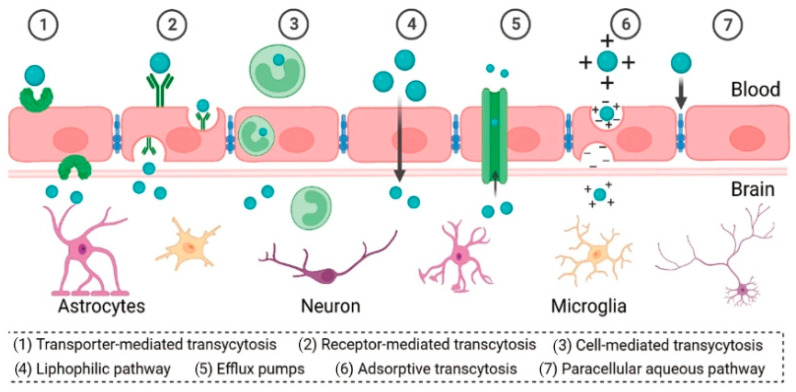
Different mechanisms for BBB crossing. Reprinted with the permission of [78].

To address this challenge, researchers are actively exploring multiple strategies to temporarily open, cross or bypass the BBB (Figure 5) [78]. One strategy involves the use of brain-targeted DDSs with specific sizes and surface property modifications. These DDSs are able to interact with transport proteins on the BBB or penetrate through the paracellular pathway, thus delivering drugs into the brain [79,80]. Another strategy is a temporary BBB opening, achieved non-invasively through methods like focused ultrasound combined with microbubble technology. This method allows for a temporary, precisely controlled breach of the barrier, enabling therapeutic drugs to reach brain tissue [81,82,83]. Researchers are also developing short peptide sequences that bind to specific receptors on the BBB [84,85]. Additionally, nasal delivery routes are being investigated for their potential to directly deliver small molecules to the brain, bypassing the BBB by utilizing the nasal-to-brain pathway [86,87,88].

Considering this array of approaches, one might inquire: How do PAMAM dendrimers facilitate the transport of drugs across the BBB to reach the brain?

### 5.1. Molecular Modeling of Dendrimers

**Figure 6:**
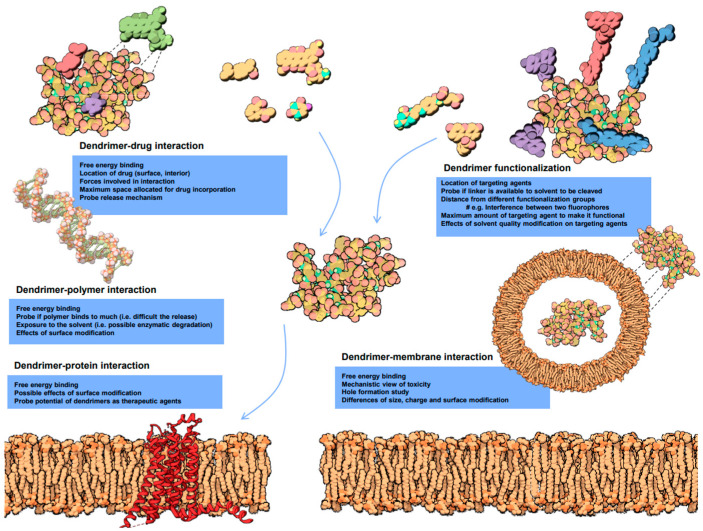
Molecular dynamics of biological interactions of dendrimers. The diagram includes dendrimer-drug interaction, dendrimer functionalization, dendrimer-polymer interaction, dendrimer-protein interaction and dendrimer-membrane interaction. # e.g., in the dendrimer functionalization part stands for an example. Reprinted with the permission of [89].

The molecular interactions can be predicted by molecular modeling strategies [89]. Understanding the complexities of molecular interactions is beneficial for optimizing the biological efficacy of dendrimers. Molecular docking studies significantly contribute to a more profound comprehension of how dendritic polymers, such as PAMAM dendrimers, interact with drugs and various biological targets (Figure 6).

### 5.2. Drug Loading Strategies for PAMAM Dendrimers

**Figure 7:**
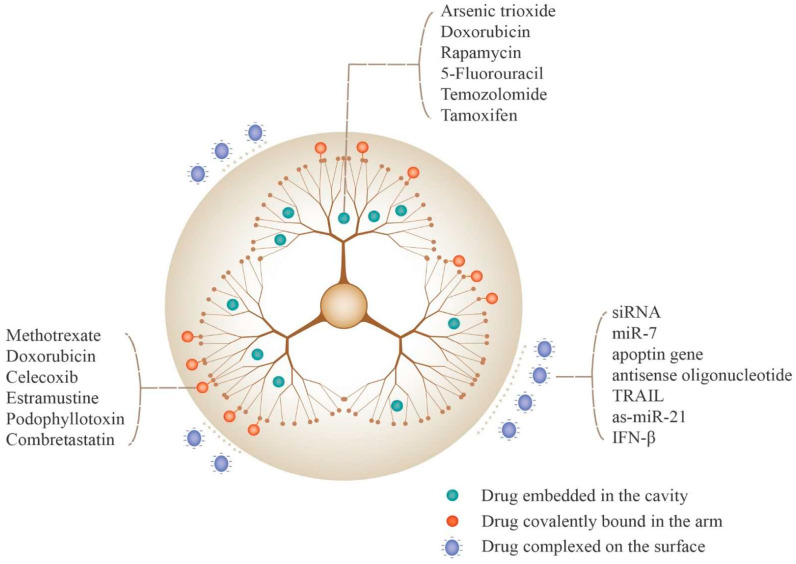
Methods for incorporating drugs to PAMAM-based DDSs and examples of drugs that can be loaded using various methods. Reprinted with the permission of [22].

Drug loading to PAMAM is affected by the structure and properties of drugs and the characteristics of PAMAM (Figure 7). There are three main methods: drug encapsulation in the core of PAMAM [90], the formation of complexes between drugs and the PAMAM surface and the covalent connection between drugs and PAMAM branches [91].

Here, we take the covalent connection as examples. In one study, microtubule inhibitor drugs, specifically estramustine and podophyllotoxin, when covalently bonded to PAMAM dendrimers, demonstrated heightened efficacy in inducing glioma cell death compared to the respective free drugs [92]. In another study, Gamage et al. synthesized a third-generation (G3) dendrimer-based conjugate with curcumin (G3-Curc) [93]. These G3-Curc nanoparticles exhibited a preferential internalization within tumor cells and, importantly, managed to accumulate specifically within the cell nuclei, indicating the potential utility of G3-Curc in the targeted treatment of brain cancers. Additionally, Li et al. designed dual-targeting DDSs based on fourth-generation PAMAM dendrimers [94]. To enhance the penetration through the BBB and promote drug accumulation specifically in glioma cells, transferrin (Tf) and tamoxifen (TAM) were selected as the targeting groups. Tf was conjugated to the outer surface, while TAM and the chemotherapy drug doxorubicin (DOX) were encapsulated within the interior. Moreover, the dendrimers were further modified with PEG.

It is known that some anticancer drugs are lipophilic. PAMAM dendrimers have large internal hydrophobic spaces and high-density surface functional groups. Hydrophobic molecules like DOX [95], sulfamethoxazole (SMZ) [96] and arsenic trioxide (ATO) [97] can be encapsulated within the inner cavity of PAMAM dendrimers through hydrophobic interactions. The encapsulation processes modify the drugs’ solubility and bioavailability, facilitating the delivery to the brain.

### 5.3. PAMAM Dendrimers as Non-Viral Vectors for Gene Delivery

Gene therapy, an innovative approach involving the replacement of abnormal or non-functional genes with normal ones [98], is utilized to treat genetic disorders through the administration of genetic substances to targeted cells, employing the following two principal methods: ex vivo and in vivo gene transfer. The ex vivo transfer entails the delivery of a gene to an organ external to the patient’s body, followed by the transplantation of the modified tissue into the recipient. The in vivo transfer introduces the gene into the target tissues directly. For efficient and safe delivery of gene into cells, appropriate vectors are necessary [99,100,101]. Gene vectors can be roughly divided into viral vectors and non-viral vectors. Viral vectors account for about 70% of the current clinical gene therapy trials. Among them, retroviruses, lentiviruses, adenoviruses and adeno-associated viruses are common types of viral vectors. Due to their excellent infectivity, viral vectors can usually achieve efficient gene transfection [102]. Nonetheless, concerns remain about the safety of viral vectors, which may elicit immune responses and cause transgene insertion mutations. In addition, there are limitations of viral vectors, such as a limited gene loading capacity, inability to deliver large genes, complexity of preparation, and so on [103]. Thus, non-viral vectors have garnered significant attention from researchers due to their notable advantages, including the substantial gene loading capacity, enhanced safety profiles and facile synthesis [104,105].

PAMAM dendrimers possess a high degree of symmetry and numerous secondary and tertiary amines, which endow them with exceptional condensation and proton buffering abilities, resulting in superior gene transfection performance [106,107]. Noteworthy, analogous to other soluble particles ranging from 10 to 100 nanometers in diameter, PAMAM dendrimers can be influenced by the EPR effect present within the tumor microenvironment [108]. Multiple studies have shown that the size of PAMAM dendrimers directly impacts their transfection capability, with partially degraded dendrimers frequently demonstrating superior transfection efficiency [109].

Below are specific instances where PAMAM dendrimers have been utilized as gene delivery vectors. Huang et al. selected PAMAM dendrimers and, by conjugating them with Angiopep-2 using bifunctional PEG, formed PAMAM-PEG-Angiopep [110]. Angiopep-2 is a ligand targeting low-density lipoprotein receptor-related protein-1 (LRP1) expressed on brain capillary endothelial cells (BCECs) and glial cells. After acquiring the PAMAM-PEG-Angiopep, they went on to develop PAMAM-PEG-Angiopep/DNA nanoparticles, aiming to achieve the targeted delivery of DNA to the brain. Similarly, chlorotoxin (CTX), known for its specificity in binding to receptors on the surface of glioma cells, was conjugated to PAMAM dendrimers via the bridging functionality of bifunctional PEG [111]. After acquiring the PAMAM-PEG-CTX complexes, researchers constructed PAMAM-PEG-CTX/DNA nanoparticles and studied the uptake efficiency of CTX in glioma cells. The distribution of PAMAM-PEG-CTX/DNA nanoparticles in the brain was significantly higher than that of other control groups, such as PAMAM/DNA nanoparticles and PAMAM-PEG/DNA nanoparticles. Also, transferrin (TF) can be conjugated by bifunctional PEG to form PAMAM-PEG-TF [112]. The PAMAM-PEG-TF group showed concentration-dependent cellular uptake and a 2.25-fold increase in brain uptake in vivo compared with the PAMAM group and PAMAM-PEG group. The PAMAM-PEG-Tf/DNA complex exhibited better transfection efficiency compared to both the PAMAM/DNA and PAMAM-PEG/DNA complexes in BCECs. With a PAMAM/DNA weight ratio of 10:1, the brain gene expression levels induced by the PAMAM-PEG-Tf/DNA complex were approximately twice as high as those observed in the PAMAM/DNA and PAMAM-PEG/DNA complexes.

### 5.4. Ligand Modifications of PAMAM for Enhanced Brain Targeting

In pursuit of enhancing the glioma-targeting precision of the PAMAM delivery system, researchers have meticulously chosen specific ligands for PAMAM modification, such as folic acid, peptides, proteins and others.

Sagir et al. developed novel folate receptor-targeted PAMAM-based DDS for photodynamic therapy [113]. The DDS feature magnetic nanoparticles were enveloped by mesoporous silica (M-MSN) outer shells. The M-MSN nanoparticles were then functionalized through the introduction of PAMAM dendrimers, with siloxane as cores and generations of PAMAM from one to three. Following this, folate molecules were grafted onto the surface of the M-MSN-PAMAM to fabricate M-MSN-PAMAM-FA. Their results demonstrated that the M-MSN-PAMAM-FA, when loaded with indocyanine green (ICG), possessed potent capabilities to induce cancer cells apoptosis. Kang C et al. used folate-PAMAM dendrimers (FA-PAMAM) to facilitate the delivery of therapeutic antisense oligonucleotides (ASODN) to C6 glioma cells [114]. The ASODN could be released from the system, resulting in decreased epidermal growth factor receptor expression in C6 glioma cells.

Arginine–Glycine–Aspartic acid (RGD) is an amino acid sequence found in a variety of proteins, particularly those involved in cell adhesion. The RGD motif serves as a critical recognition signal for the interaction between extracellular matrix proteins and integrins (cell surface receptors), playing a significant role in processes such as cell adhesion, migration and signaling. Zhu et al. fabricated cyclized RGD-modified PEG-PAMAM and conjugated with doxorubicin (DOX) via an acid-sensitive cis-aconityl linkage, which was termed RGD-PPCD [115]. RGD-PPCD significantly enhanced the targeting of DOX to tumors by specifically recognizing and binding to integrin receptors on the surface of tumor cells. RGD-PPCD can accurately regulate the release of DOX under the weak acidic lysosomal environment in tumor cells through acid-sensitive cis-aconityl. Compared with the free DOX solution, RGD-PPCD not only significantly prolonged the half-life of DOX, but also achieved high concentration aggregation in brain tumor tissues. Shi X et al. modified PAMAM-PEG with an internalized RGD (iRGD) and a 12-amino acid TGN peptide (TGNYKALHPHNG), and encapsulated trioxide arsenate (ATO) derived from traditional Chinese medicine into this system [116]. The results showed that the encapsulation efficiency of ATO was about 71.92% ± 1.17%. The iRGD/TGN co-modified group exhibited longest survival rate compared to other groups. Sharma et al. precisely and systematically modified the surfaces of PAMAM dendrimers with glucose, mannose or galactose moieties, aiming to target the overexpressed sugar transporters in glioma [24].

## 6. Concluding Remarks

The main treatments for glioma include surgical intervention, radiotherapy and chemotherapy [117]. However, there are many difficulties in these treatments. The strategy of brain-targeted DDSs represents a promising adjunct in glioma therapy, alleviating difficulties in traditional treatments. PAMAM dendrimers with particle sizes between 10 nm and 100 nm are suitable for developing as DDSs to target brain lesions. The interiors of PAMAM dendrimers are composed of branches which can be used to load drugs with low solubility, such as paclitaxel, doxorubicin and other lipophilic anticancer drugs. By loading to PAMAM, those lipophilic drugs can be transformed to amphiphilic drugs, enabling improved biocompatibility, permeability and stability. The inherent cytotoxicity associated with the cationic surface groups of PAMAM dendrimers necessitates strategic modifications. Surface engineering with targeted peptides not only attenuates cytotoxic effects but also confers brain-homing capabilities. Additionally, in recent years, by decorating biomembranes onto PAMAM dendrimers, PAMAM dendrimers can be equipped with biomimetic properties. For instance, coated with erythrocyte membranes, the systemic circulation time of PAMAM dendrimers can be prolonged like natural erythrocytes.

As DDS continue to evolve, new ligands and combinations are being developed, leading to constant updates in modifying PAMAM dendrimers. Numerous experimental studies have provided valuable insights into brain-targeted PAMAM-based DDSs. Meanwhile, the exploration of PAMAM-based DDSs in selective drug delivery and accurate diagnostic applications is ongoing. Despite ongoing advancements in PAMAM-based DDSs, remarkably few have successfully navigated the path from the bench to clinical trials. To bridge this translational gap, further efforts are required to establish standardized protocols.

## Data Availability

Not applicable.
